# The quality of Tibetan sheep meat from pastures was synergistically regulated by the rumen microbiota and related genes at different phenological stages

**DOI:** 10.3389/fvets.2024.1484175

**Published:** 2025-01-07

**Authors:** Xiaowei Chen, Yuzhu Sha, Xiu Liu, Yanyu He, Wenhao Li, Liangwei Yao, Jiqing Wang, Wenxin Yang, Qianling Chen, Min Gao, Wei Huang, Bin Ma

**Affiliations:** ^1^Gansu Key Laboratory of Herbivorous Animal Biotechnology, College of Animal Science and Technology, Gansu Agricultural University, Lanzhou, China; ^2^School of Fundamental Sciences, Massey University, Palmerston North, New Zealand; ^3^Academy of Animal Science and Veterinary Medicine, Qinghai University, Xining, China; ^4^Zhangye City Livestock Breeding and Improvement Workstation, Zhangye, China

**Keywords:** Tibetan sheep, meat quality, rumen microbiota, phenology period, gene expression

## Abstract

Meat quality is a key indicator of meat performance in ruminants, and its mechanism and regulation are also key to ruminant research. Studies have shown that animal meat quality is related to the gut microbiota. In this study, RT-qPCR and 16S omics were employed to assess meat quality and intestinal microbiota. The objective was to investigate the influence of seasonal variations on the meat quality of Tibetan sheep ewes by examining the rumen microflora, meat quality attributes, and associated gene expression profiles over three distinct months: May, August, and December.The results indicate that muscle tenderness was significantly greater (*p* < 0.001) in the grass period than in the regrowth and dry grass periods and was highest in the longest dorsal muscle. The cooking rate of the foreleg muscle was significantly greater (*p* < 0.05) than that during the regrowth and dry grass periods, and the pH24h significantly differed (*p* < 0.05) across the different seasonal periods. The crude protein content of the longest back muscle and the foreleg muscle was significantly greater (*p* < 0.001) than that of the wither and grass stages during the regrowth period and slightly decreased during the grass stage. The crude fat and crude ash contents of the three groups differed significantly, and the fat content during the grass stage was significantly (*p* < 0.05) greater than that during the regrowth stage and the wither stage. Expression analysis of genes related to meat quality revealed that the expression of the ADSL gene was significantly greater (*p* < 0.05) in the anterior and posterior leg muscles during the grass period than during the regrowth and wilting periods, whereas the expression of the FABP3 gene was lower than that during these two periods. Correlation analysis revealed that Rikenellaceae_RC9_gut_group was significantly positively correlated (*p* < 0.05) with shear forceand cooked meat percentage and significantly negatively correlated (*p* < 0.05). Ruminococcus and Butyrivibrio were significantly positively correlated (*p* < 0.05) with CAST and highly significantly positively correlated (*p* < 0.05). In conclusion, meat quality during different seasons is regulated by the rumen microbiota and their associated genes.

## 1 Introduction

The Tibetan Plateau is the highest plateau on Earth and is frequently designated the “Roof of the World” or the “Third Pole.” This area has distinctive climatic features, including extreme coldness, a lack of oxygen, and intense ultraviolet radiation. This singular ecological setting has engendered exceptional adaptive traits among native organisms. Alpine vegetation faces severe constraints due to this rigorous environment, resulting in a brief growing season marked by seasonal fluctuations in nutrient composition (1). The greening phase (early April to early May), vegetative phase (June–September), and dormant phase (October–March) of alpine forage mirror variations in temperature (2). Vegetation during vegetative phases is diverse, rich in nutrients, and has elevated levels of proteins and carbohydrates (3), whereas during dormant or greening stages, the underdeveloped state leads to diminished nutritional value and quality (4). Phenological shifts within plateau ecosystems significantly influence plant life, cattle development, and the intestinal microbiota (5).

Tibetan sheep, the original breed bred at an altitude of more than 2,000 m above sea level on the Qinghai–Tibet Plateau, serve as the primary livestock breed and genetic resource in this region. It is also a cornerstone industry for local herdsmen’s livelihoods and plays a crucial role in the ecosystem of the plateau (6). Compared with other sheep breeds, Tibetan sheep exhibit resistance to roughage feeding, exceptional meat production performance, and superior adaptation to the challenging ecological conditions of intense cold, hypoxia, and limited nutrition on the Qinghai–Tibet Plateau (7). Tibetan sheep meat is characterized by its high protein content, low fat content, and high amino acid and fatty acid contents (8). The quality of mutton is influenced by various factors, such as breed variety, sex, age, environment, feed nutrition and additives (9). Changes in these factors lead to variations in mutton characteristics. Among them are alterations in muscle nutrient composition—particularly changes in fat and protein content—which directly impact meat quality and nutritional attributes (10). The nutritional composition of feeds at different phenological stages can influence microbial metabolic pathways or gene expression. This modulation affects the physical and chemical properties of meat, which ultimately results in differences in meat quality (8, 11).

The rumen of ruminants is an ideal environment for anaerobic microorganisms. Rumen microorganisms are essential for ruminant health. Ruminants rely on the fermentation of microorganisms in the rumen to digest plant fiber and convert nutrients that cannot be used directly into animal protein for use by the host (12). The diet of ruminants is mainly forage-based, and the nutritional components of forage are closely related to the composition and abundance of rumen microorganisms (13). Rumen microorganisms play an important role in the digestion and utilization of forage. Changes in the abundance and composition of the flora can lead to changes in feed digestibility and rumen function (14), which in turn affect the quality of the meat. Therefore, the rumen microbial flora has an important influence on the meat quality of ruminants. For example, herbal tea residue, as a source of functional roughage, affects rumen fatty acid composition and muscle glycolipid metabolism by altering the microbial composition of the rumen to improve meat quality in steers (15). In addition, different proportions of roughage in the diet can lead to changes in rumen fermentation and can have potentially beneficial effects on meat quality (16). The addition of microbial fermented diets can improve growth performance and meat quality in beef cattle; but too high a proportion may reduce the negative impact of rumen energy metabolism on growth performance (17).

These changes may affect meat quality by altering the rumen environment. Until now, it has not been clear that the dynamics of the rumen microbiome of Tibetan sheep grazing during different seasonal periods correlate with their growth performance, intestinal health and meat quality when the rumen environment changes due to changes in the nutrient composition of the pasture. Therefore, in this study, we identified rumen microorganisms in samples via 16S rDNA amplicon sequencing to elucidate the differences in rumen microbial diversity as well as the changes in species composition in Tibetan sheep during different phenological periods. The correlations between differential microorganisms in the rumen and meat quality-related genes, as well as the correlations between these factors and meat quality, provide a reference for improving the meat quality of Tibetan sheep grazing during different seasonal periods.

## 2 Materials and methods

### 2.1 Experimental design and sampling

All studies involving animals were carried out in accordance with the regulations of the Administration of Affairs Concerning Experimental Animals (Ministry of Science and Technology, China; revised in June 2004), and sample collection protocols were approved by the Livestock Care Committee of Gansu Agricultural University (Approval No. GAU-LC-2020-27). Eighteen healthy 1-year ± 1-month-old disease-free grazing Tibetan sheep with similar body weights of 30.23 ± 4.04 kg were randomly selected from the same pasture in Yeniugou Township, Qilian County, Qinghai Province, and marked with a digital ear tag, and they were randomly assigned to three groups, Each group contains 6 sheep. According to the plant phenology of the Qinghai–Tibet Plateau, Tibetan sheep with similar body weights were divided into the following three groups: (1) the regreening period group (Fr, *n* = 6); (2) the green-grass period group (Qr, *n* = 6); and (3) the withered grass period group (Kr, *n* = 6). All Tibetan sheep grazed on the natural grassland in Yeniugou Township, Qilian County, Qinghai Province (E101°02′, N36°05′, altitude approximately 3,200 m). The Tibetan sheep were provided ad libitum access to food and water throughout the duration of the experiment. The vegetation was characteristic of alpine meadows. In the study area, Tibetan sheep typically grazed in an all-grazing system, with alpine meadows serving as their sole forage. The species composition during the greening period was predominantly composed of cold- and drought-tolerant plants, including *Stipa* and *Leymus secalinus*. The grass period was characterized by a diverse array of grass species, including Tibetan grass (*Kobresia pygmaea*), alpine songgrass (*Kobresia humilis*), and a variety of grasses and sedge plants. The species composition of the dry grass period was characterized by the prevalence of hardy plants, including alpine tarragon and needle fescue. Throughout the experimental period, a series of measures were implemented to ensure the stability of the experimental conditions and the reliability of the results. First, pesticides and fertilizers were avoided in the grassland to prevent the effects of chemicals on the health of Tibetan sheep and the rumen microbiota. Furthermore, to minimize grazing competition and the potential for cross-contamination, the grazing of other livestock on the experimental grassland was strictly controlled, and access to the area by nonexperimental Tibetan sheep and other domestic animals was restricted. Concurrently, the vegetation cover and soil conditions of the grasslands were subjected to periodic monitoring to ascertain their overall health and the extent of grazing pressure.

In accordance with the ethical approval requirements of the Ethics Committee and the local traditional slaughter sampling method, immediately after cervical bloodletting and slaughter of the Tibetan sheep in each of the three seasonal periods, the gastrointestinal organs were removed, the rumen contents were collected, and approximately 50 mL of ileal contents were collected from each sheep, dispensed into cryopreservation tubes, rapidly frozen in liquid nitrogen tanks, and returned to the laboratory for preservation at −80°C for subsequent 16S rRNA analyses. Samples of the longest dorsal muscle (the muscle between the second thoracic vertebra and the second lumbar vertebra), the anterior hamstring (triceps brachii) and the posterior hamstring (biceps femoris) were then taken, placed in cryopreservation tubes, rapidly frozen in a tank of liquid nitrogen and returned to the laboratory for preservation at −80°C for subsequent RNA extraction. Subsequently, 500 g of dorsal longest muscle, anterior leg muscle and posterior leg muscle samples were collected, returned to the laboratory and placed in a refrigerator at −20°C for subsequent determination of meat quality. Eighteen 50 cm × 50 cm quadrats were randomly selected from grasslands grazed by Tibetan sheep. Six quadrats were sampled from each period. Edible pastures were stored and dried in an oven at 65°C until a constant mass was reached and then sieved at 1 mm.

### 2.2 Measuring indicators and methods

#### 2.2.1 Forage nutrient content

The nutrient contents of a total of 18 grass samples were analyzed, with three technical replicates established for each biological sample. The Association of Official Analytical Chemists (AOAC) method (18) was employed for the determination of dry matter (DM) and ash in grasses. The dry matter (DM) content was determined by drying the samples in a blower oven at 105°C for 4 h. The DM content was also determined by drying the samples in a blower oven at 550°C for 4 h. The ash content was analyzed by complete combustion of the samples in a muffle furnace at 550°C for 6 h. The DM content was determined via the Association of Official Analytical Chemists (AOAC) method. The nitrogen content was determined via a protein tester (Shanghai Ruiphan International Trading Co., Ltd., Model KJELTEC8200) in accordance with the Kjeldahl method. The crude protein (CP) content was subsequently calculated as *N* × 6.25. The crude fat content was determined with a fully automated Soxhlet fat extractor (Shanghai Ruiphan International Trading Co., Ltd., Model SOXTEC2050). The analysis of neutral detergent fiber (NDF) and acid detergent fiber (ADF) contents was conducted in accordance with the methodology proposed by Van Soest et al. (19). Six quadrats per group.

#### 2.2.2 Detection of the edible quality and conventional nutrients of meat

After thawing, the meat quality of the Tibetan sheep samples was determined via conventional methods, including pH at 1 h and 24 h, shear force, cooked meat percentage and water loss. pH was determined by fully immersing the electrode head of a calibrated pH meter (TESTO 205, DeTu Instrument Co., Ltd., TESTO 205) into the meat samples (avoiding fat and fascia at the same time) until it reached the center of the meat samples; after stabilization, the electrode remained in place for >5 min to read the values (20), and the average of three parallel measurements was taken. The shear force was determined via a constant-temperature water bath (Jiangsu Jintan Ronghua Instrument Manufacturing Co., Ltd., Type HH-2), and the shear force was measured by cutting in a direction perpendicular to the muscle fibers (meat sample size: 2 cm × 1 cm × 1 cm) with a tenderness meter (21). A representative lamb sample of approximately 200 g was selected, and its initial weight W1 was recorded. The sample was placed in the steamer drawer of an aluminum steamer with boiling water on a 2,000 W electric cooker for 45 min, removed and left to cool for 30–45 min or hung indoors in a cool, sheltered place, weighed after 30 min and recorded as W2, and the percentage of cooked meat = W2/W1 × 100%. Water loss was determined according to the method of Honikel (22). All the experiments were repeated 3–5 times to obtain the means.

The crude ash, crude fat, crude protein contents of the meat samples were determined according to specified meat testing standards to reflect the nutritional quality of the meat. The crude protein content was determined via a Kjeldahl nitrogen analyzer (Shanghai Ruifen International Trading Co., Ltd., KJELTEC8200), the crude fat content was determined via an automatic Soxhlet fat extraction leachometer (Shanghai Ruifen International Trading Co., Ltd., SOXTEC2050), the crude ash content was determined via a precision blotting machine, Ltd., SOXTEC2050, and the crude ash content was determined using a precision blast drying oven (Shanghai YIHE Scientific Instrument Co., Ltd., BPG-9240A); for moisture content, the samples were heated in an oven at 150 ± 2°C until the weight was constant, and the meat samples were calcined in a crucible at 550°C for 4 h to determine the ash content. The determination was performed according to the AOAC method (23).

#### 2.2.3 Analysis of mRNA levels in muscle tissue

Total RNA was extracted from muscle tissue following the protocol of the TRIzol kit (Ambion). The concentration and purity of the total RNA were determined via a NanoDrop One Ultra-Micro UV Spectrophotometer (Thermo, United States). The RNA was subsequently reverse transcribed into cDNA using the EvoM-MLV Reverse Transcription Kit (Acres Bio, Hunan, China). The reverse transcribed cDNA was stored at −20°C for further use in detecting the expression of relevant genes. The primers for the *CAST*, *MSTN*, *FABP3*, *ADSL*, *LPL* and *SCD* genes were designed with Primer 5.0 software (*β-actin* served as the internal reference gene) and synthesized by Beijing Auclean Ding Sheng Biotechnology Co., Ltd. The primer information is shown in Table 1.

**Table 1:** Primer for detection of meat quality related genes.

Gene	Primers sequence (5′ → 3′)	Length/bp	Gene accession no.
*β-actin*	F: AGCCTTCCTTCCTGGGCATGGA	113	NM_001009428.3
	R: GGACAGCACCGTGTTGGCGTAA		
*MSTN*	F: AACAGCGAGCAGAAGGAAAA	145	NM_001009788.1
	R: GGTTAAATGCCAACCATTGC		
*CAST*	F: CGAGATTTCCGGTGGTGGAA	122	NM_001009394.1
	R: GCTTGGATTCAACTGGCACG		
*LPL*	F: TGGAGTGACGGAATCTGTGG	150	NM_001267884.2
	R: ACGTTGGAGTCTGGTTCCCT		
*FABP3*	F: GTCTCTTTCCCGACCTAGCC	118	XM_004007004.5
	R: TAGCAAAACCGACACCGAGT		
*ADSL*	F:CGCTTGCTTCCCGTTATGC	73	AJ_001048
	R: CGCCAGGTCCGGAATTTGTA		
*SCD*	F:CCCAGCTGTCAGAGAAAAGG	115	NM_001009428.3
	R: GATGAAGCACAACAGCTTGT		

Real-time fluorescence quantitative PCR was used to detect the relative expression of meat quality-related genes in three parts of muscle tissue according to the instructions of the ChamQ Universal SYBR Green PCR Kit Q711002/03 Vazyme. The total reaction volume was 20 μL. The entire reaction process was carried out on an Applied Biosystems Quant Studio6 Flex real-time PCR instrument (Thermo Fisher Scientific, MA, United States). The reaction conditions were predenaturation at 95°C for 30 s, denaturation at 95°C for 5 s, annealing at 60°C for 30 s, and extension at 72°C for 30 s. A total of 40 cycles of data analysis were performed using the $2^{-\Delta \Delta C_{T}}$ method (24).

#### 2.2.4 Analysis of microbial diversity in the rumen across different phenological stages

The bacterial DNA extraction kit MN NucleoSpin 96 Soi Omega Shanghai China Rumen microbiome DNA was extracted to encode bacterial ribosomal RNA. The conserved regions of the nucleotides were mainly the 16S region. The forward primer 38F: 5′-ACTCCTACGGGAGGCAGCA-3′ and the reverse primer 806R: 5′-GGACTACHVGGTWTCTAAT3′ were used to amplify the V3–V4 region amplicon library of the 16S rRNA gene highly variable region via PCR amplification. End-to-end sequencing was performed at Beijing Biomarker Technology Co., Ltd., Beijing, China, 2 × 250, and the reads from the original sequencing data were double-end spliced via FLASH Version 1.2.11 to obtain the original label data. Trimmomatic 0.33 was used for quality filtering to obtain high-quality label data to identify the chimeric sequence, and QIIME2 Version 2020.6.0 was removed to obtain the final valid data and then cleanly read for feature classification. The ASV amplicon sequence variant was output by DADA2 (25) to filter the ASV circles less than 2 in all samples. On the basis of the naive Bayesian classifier in QIIME2 (26), the SILVA database (27) was used to classify ASVs, and the label confidence threshold was set at 70%. QIIME2 2020.6 software (26) was used to evaluate the alpha diversity indices (ACE, Chao1, Simpson and Shannon indices) of the samples. The alpha diversity index was tested for a normal distribution prior to ANOVA via Shapiro–Wilk (SW) analysis at different taxonomic levels (phylum, genus, family and species), and the principal coordinate analysis (PCoA) diagram of the corresponding distance samples was obtained via the SILVA database Version 138.1 (27) on the basis of the distance matrix algorithm. PICRUSt software was used to perform KEGG Kyoto Encyclopedia of Genes and Genomes and COG Clusters of Orthologous Groups of protein function prediction on 16S sequencing data.

### 2.3 Statistical analysis

SPSS 26.0 software (IBM Corporation, Armonk, NY, United States) was used for statistical analysis. One-way ANOVA was used to analyze the rumen bacteria of the feed and meat nutrients. Duncan’s test was used for multiple comparisons of means. *p* < 0.05 indicates a significant difference; *p* ≥ 0.05 indicates no significant difference; and 0.05 < *p* < 0.10 indicates a significant trend. To determine the correlation between meat quality and meat quality genes and the correlation with rumen microbial composition, the Pearson correlation coefficient was used to create a visual correlation map.

## 3 Results

### 3.1 Nutritional components of forage grass during different phenological periods

The nutrient composition of the forage grass in each phenological period is shown in Table 2. The DM and NDF contents during the withering period were significantly greater than those during the returning-green period and grass period (*p* < 0.01). However, the CP and CF contents in the grass period were greater than those in the returning-green period and the grass period and significantly greater than those in the other two periods (*p* < 0.01). There was no significant difference in ADF among the three periods (*p* > 0.05).

**Table 2:** Conventional nutrient content of forage in regreening stage and grassy stage (dry matter basis) %.

Items	Regreen period	Green period	Hay period
DM	93.25 ± 0.14^b^	89.59 ± 0.64^c^	94.19 ± 0.23^a^
CP	6.45 ± 0.16^b^	19.02 ± 0.69^a^	5.71 ± 0.16^c^
EE	2.77 ± 0.16^b^	3.41 ± 0.26^a^	0.64 ± 0.03^c^
ADF	29.05 ± 2.22	27.20 ± 4.92	33.20 ± 2.60
NDF	44.97 ± 0.74^c^	55.52 ± 1.24^b^	58.44 ± 0.17^a^

Different lowercase letters indicate significant differences with *p* < 0.05.

### 3.2 Meat quality and nutritional components of Tibetan sheep in different phenological periods

As shown in Table 3, the water loss rate of the hind leg muscle was significantly greater (*p* < 0.05) in the regreening period than in the wilting and green-grass periods. The tenderness of the three parts was significantly greater (*p* < 0.001) in the grass stage than in the regreening and withering stages, and the cooked meat percentage of the foreleg muscle was significantly greater (*p* < 0.05) than that in the withering and regreening stages. The pH at 1 h was 5.99–8.22 during the different periods, which was greater during the returning-green period than during the grass and hay periods. The pH at 24 h for the different periods ranged from 5.71–7.45. The lowest nutritional components in different parts of the hay period included moisture, crude protein, crude fat and ash.

**Table 3:** The objective of this study is to examine the meat quality parameters of Tibetan sheep at different seasonal periods.

Items	Parts	Regreen period	Green period	Hay period	*p*
Water loss capacity/%	Longissimus dorsi muscle	0.33 ± 0.08	0.23 ± 0.02	0.26 ± 0.16	0.315
	Musculi cruralis anterior	0.23 ± 0.08	0.25 ± 0.01	0.343 ± 0.14	0.116
	Muscle of hind leg	0.33 ± 0.04^ab^	0.24 ± 0.01^b^	0.22 ± 0.09^b^	0.009
Shear force (N)	Longissimus dorsi muscle	46.51 ± 0.69^b^	75.21 ± 1.36^a^	43.68 ± 0.86^c^	<0.001
	Musculi cruralis anterior	37.36 ± 0.51^c^	55.81 ± 2.41^a^	47.17 ± 2.81^b^	<0.001
	Muscle of hind leg	60.29 ± 0.75^a^	59.05 ± 7.23^a^	41.40 ± 0.84^b^	<0.001
Cooked meat rate/%	Longissimus dorsi muscle	0.48 ± 0.08	0.53 ± 0.06	0.55 ± 0.12	0.404
	Musculi cruralis anterior	0.56 ± 0.07^ab^	0.65 ± 0.10^a^	0.45 ± 0.10^b^	0.05
	Muscle of hind leg	0.55 ± 0.07	0.50 ± 0.09	0.56 ± 0.10	0.753
pH_1h_	Longissimus dorsi muscle	7.19 ± 0.46^a^	6.20 ± 0.21^b^	6.28 ± 0.03^b^	0.02
	Musculi cruralis anterior	7.84 ± 0.38^a^	6.20 ± 0.17^b^	6.32 ± 0.13^b^	<0.001
	Muscle of hind leg	7.60 ± 0.58^a^	6.49 ± 0.14^b^	6.41 ± 0.03^b^	0.01
pH_24h_	Longissimus dorsi muscle	7.08 ± 0.40^a^	6.27 ± 0.10^b^	5.72 ± 0.01^c^	<0.001
	Musculi cruralis anterior	7.02 ± 1.06^a^	6.29 ± 0.03^ab^	5.90 ± 0.01^b^	0.02
	Muscle of hind leg	6.81 ± 1.50	6.34 ± 0.16	5.87 ± 0.04	0.21
Moisture/%	Longissimus dorsi muscle	73.52 ± 1.31^b^	72.53 ± 0.28^b^	76.24 ± 0.39^c^	<0.001
	Musculi cruralis anterior	76.92 ± 1.75^a^	75.74 ± 0.16^ab^	74.85 ± 1.15^b^	0.03
	Muscle of hind leg	75.71 ± 1.49^ab^	74.99 ± 1.00^b^	76.64 ± 0.34^a^	0.05
Crude protein/%	Longissimus dorsi muscle	22.55 ± 0.35^a^	20.21 ± 0.24^b^	19.59 ± 1.76^b^	0.02
	Musculi cruralis anterior	21.23 ± 2.19^a^	19.38 ± 0.28^b^	14.94 ± 0.57^c^	<0.001
	Muscle of hind leg	20.60 ± 0.68^ab^	20.05 ± 0.34^b^	20.75 ± 0.40^a^	0.07
Crude fat/%	Longissimus dorsi muscle	2.56 ± 0.05^ab^	3.01 ± 0.42^a^	2.22 ± 0.55^b^	0.01
	Musculi cruralis anterior	2.85 ± 0.11^b^	3.41 ± 0.15^a^	2.54 ± 0.19^c^	<0.001
	Muscle of hind leg	3.74 ± 0.12^a^	3.93 ± 0.27^a^	2.80 ± 0.58^b^	<0.001
Ash/%	Longissimus dorsi muscle	1.08 ± 0.01^b^	1.08 ± 0.03^b^	2.08 ± 0.14^a^	<0.001
	Musculi cruralis anterior	1.00 ± 0.03^b^	1.04 ± 0.02^b^	1.65 ± 0.10^a^	<0.001
	Muscle of hind leg	1.06 ± 0.01^b^	1.16 ± 0.01^b^	1.97 ± 0.023^a^	<0.001

pH_1h_ and pH_24h_ indicate the pH values of muscle cross-sections 1 h and 24 h after slaughter. Different lowercase letters in the same column indicate significant differences between different phenological stages at *p* < 0.05 level.

### 3.3 The expression of genes related to the meat quality of Tibetan sheep in different phenological periods

**Figure 1:**
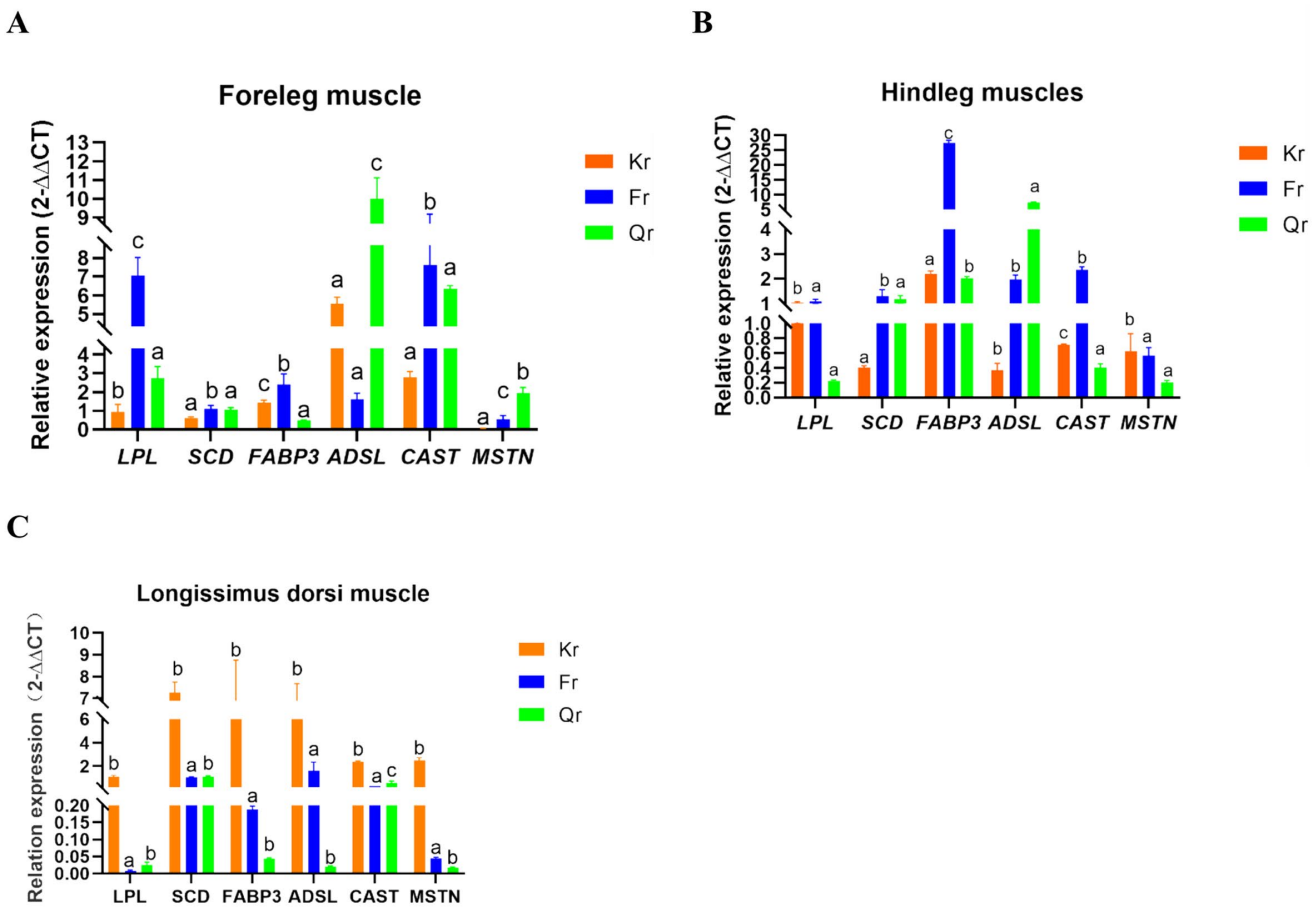
Analysis of gene expression in muscle tissue of Tibetan sheep at different phenological stages. **(A)** Dorsal longest muscle gene expression analysis. **(B)** Foreleg muscle gene expression analysis. **(C)** Hind leg muscle gene expression analysis. Different lower case letters indicate a significant difference between the age groups at the *p* < 0.05 level.

As shown in Figure 1, there were significant differences in the expression of muscle quality-related genes in Tibetan sheep during the three phenological periods (*p* < 0.05). Among these genes, the expression of the *SCD* gene in the longissimus dorsi muscle was highest in the grass period, and the expression of the *CAST* gene was significantly greater than that in the grass period and the grass period (*p* < 0.05). In the foreleg muscle, the level of *LPL FABP3* was significantly greater in the regrowth period than in the grassy period and the regrowth period (*p* < 0.05). Among these genes, *ADSL* had the highest expression in the hind leg muscle during the grassy period and the lowest expression in the longissimus dorsi muscle. The expression of *CAST* and *FABP3* in the hind leg muscle was significantly greater in the regreening period than in the grassy period and the grassy period (*p* < 0.05).

### 3.4 Characteristics of the rumen microbial flora in Tibetan sheep at different phenological stages

**Figure 2:**
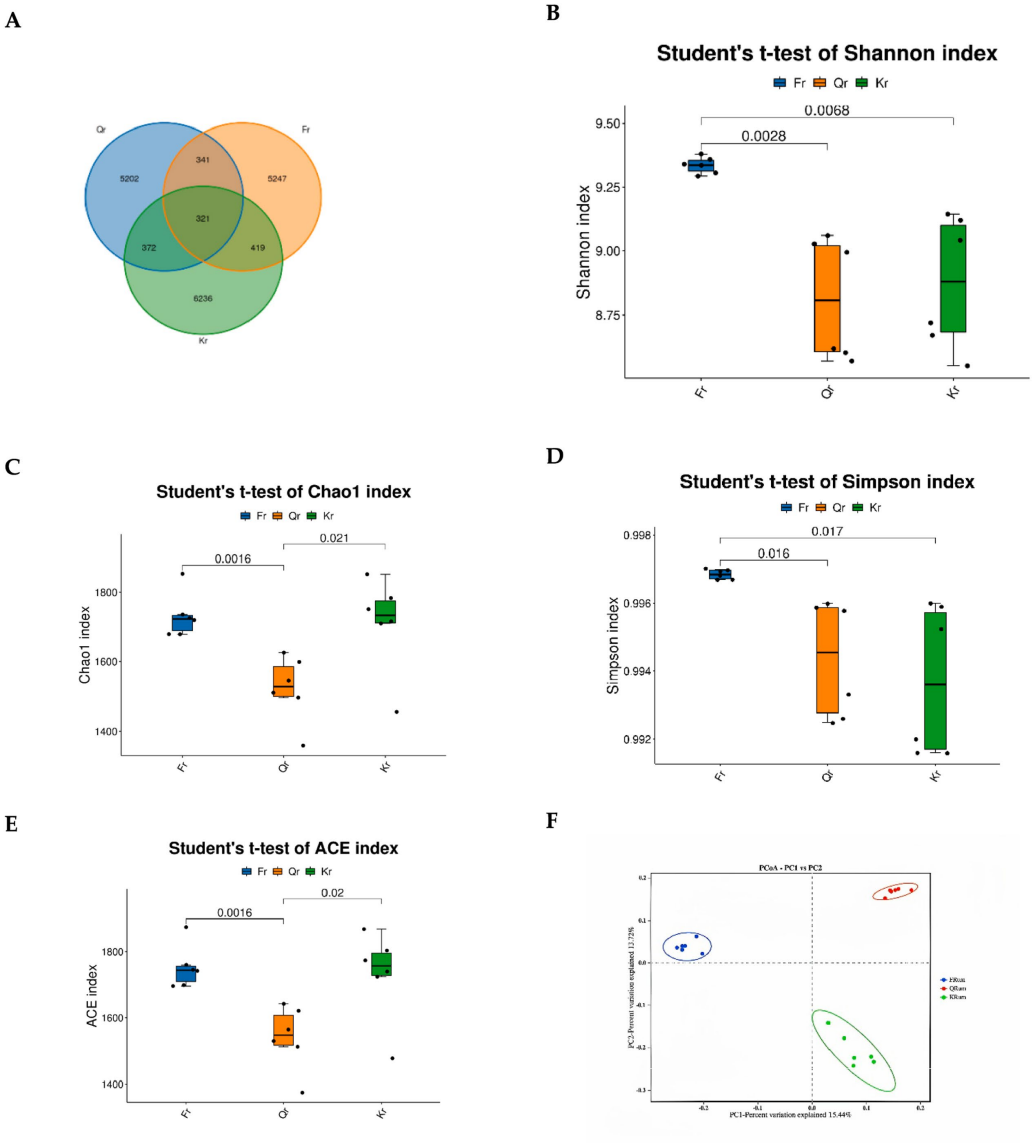
Diversity analysis of rumen flora in different seasonal periods. **(A)** QTU. **(B)** Shannon. **(C)** Chao1. **(D)** Simpson. **(E)** ACE. **(F)** PCoA. Fr represents regreening period group. Qr represents green grass group. Kr represents withered grass period group.

In this study, a total of 1,440,906 pairs of reads were obtained after quality control splicing of the reads. A total of 1,318,093 clean reads were generated after quality control splicing of the reads. Each sample produced at least 72,691 clean reads, and an average of 73,227 clean reads were generated. The Good’s coverage of all samples was greater than 0.99. A total of 14,494 ASVs and 5,247 samples were analyzed in the study, 5,202, and 6,236 specific ASVs were observed in the green-grass stage and the wilted-grass stage, respectively (Figure 2A). Alpha diversity analysis revealed that the ACE, Chao1, Shannon, and Simpson indices of rumen microorganisms in Tibetan sheep were significantly different (*p* < 0.05) (Figure 2B–E). The Shannon and Simpson indices of the returning-green period were significantly greater than those of the grass period and the withered-grass period (*p* < 0.05). The results revealed that the abundance of rumen microbial species in the returning-green period was significantly greater than that in the grass period and the withered-grass period, whereas the Chao1 and ACE indices were significantly greater than those in the grass period (*p* < 0.05). The Chao1 and ACE indices of the grass period were significantly lower than those of the wilted-grass period (*p* < 0.05). PCoA revealed that the clouds of the returning-green period and the withered-grass period were separated from each other. The distance of the rumen microorganisms in the three phenological periods was wide, indicating that there were significant differences in the rumen microbial species among the three groups (Figure 2F).

**Figure 3:**
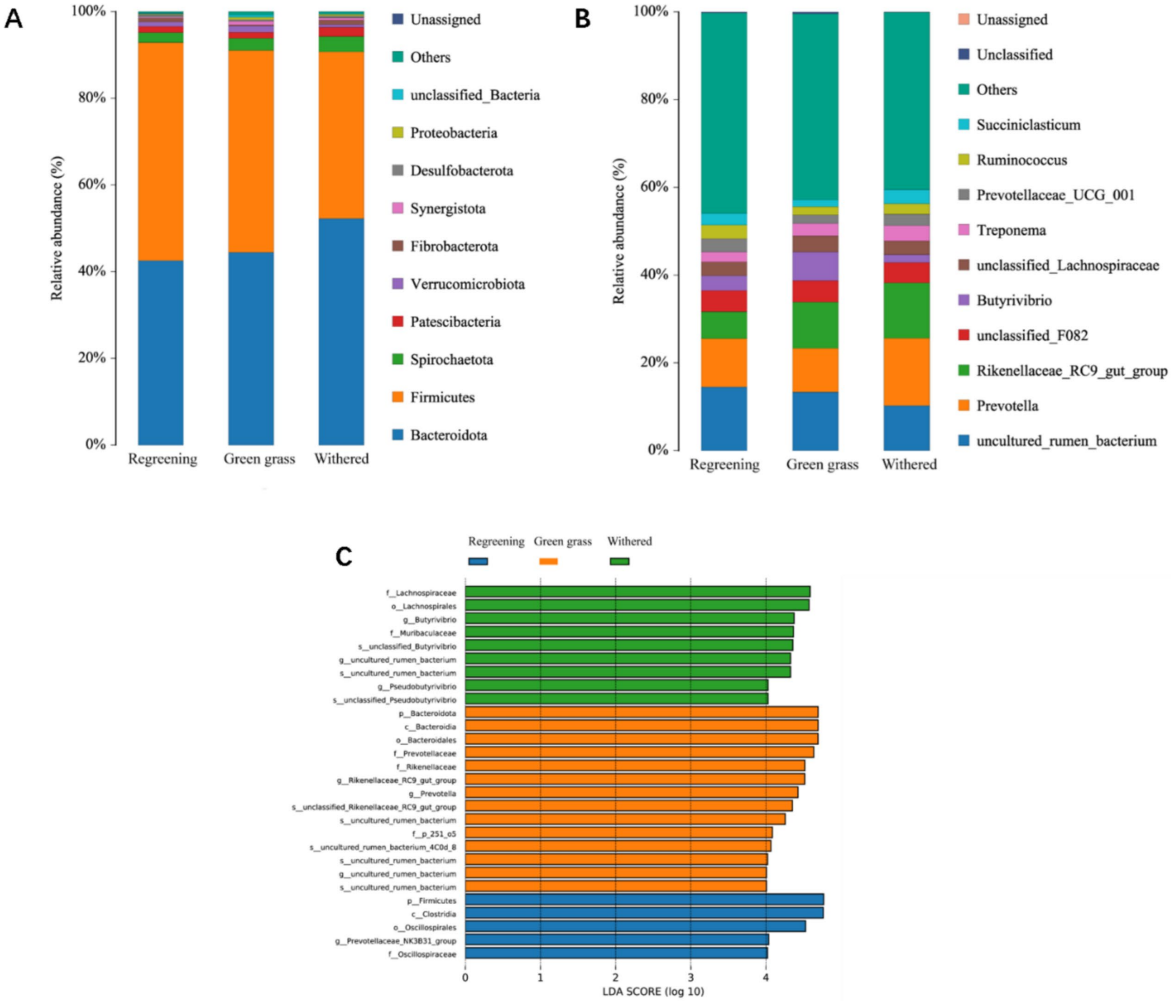
Analysis of microbial composition of Tibetan sheep at different phenological stages. **(A)** Microbial composition of phyla levels. **(B)** Microbial composition of genus levels. **(C)** The linear discriminant analysis (LDA) effect histogram between groups showed that the LDA score (log10) was greater than 4. Fr stands for the rejuvenation group. Qr stands for the grassy group. Kr stands for the wilting group.

At the taxonomic level, a total of 28 phyla, 59 classes, 145 orders, 257 families, 458 genera, and 546 species were detected. At the phylum level, Bacteroidetes, Firmicutes, Spirochaetota, Patella bacteria, and Patescibacteria were the dominant phyla, with relative abundances greater than 1% (Figure 3A). The relative abundances of Bacteroidetes and Firmicutes were highest in the different phenological periods, accounting for more than 90% of the total abundance. The abundances of Bacteroidetes and Spirochaetota were greater in the grassy period than in the returning-green period and grassy period. The abundance of Firmicutes was lower at the genus level. The *Prevotella_1 Rikenellaceae_RC9_gut_*group was the dominant genus in the different phenological periods. A total of 28 differences were found in 458 genera (*p* < 0.05). Among the top 10 species, the abundances of *Prevotella_1* and *Rikenellaceae_RC9_gut_group* were greater in the grassy period than in the grassy period and the returning-green period. Compared with that in the returning-green period and the grassy period, the abundance of *Butyrivibrio* in the grassy period was greater than that in the returning-green period. In addition, this study also identified many *Treponema* species in the genus *Succiniclasticum Prevotellaceae_UCG_001* (Figure 3B). LEfSe analysis revealed that 28 genera were identified by discriminant analysis (Figure 3C) among the three groups of samples. *p__Firmicutes*, *c__Clostridia*, *o__Oscillospirales*, *g__Prevotellaceae_NK3B31_group*, and *f__Oscillospiraceae*, *lospiraceae* were enriched in the 5 genera in the replanting period. There were 14 genera, *p__Bacteroidota*, *c__Bacteroidia*, *o__Bacteroidales*, *f__Prevotellaceae*, *f__Rikenellaceae*, *g__Rikenellaceae_RC9_gut_group*, *g__Prevotella*, *s__unclassified_Rikenellaceae_RC9_gut_group*, *s__uncultured_rumen_bacterium*, *f__p_251_o5*, *s__uncultured_rumen_bacterium_4C0d_8*, *s__uncultured_rumen_bacterium*, *g__uncultured_rumen_bacterium*, and *s__uncultured_rumen_bacterium*, in the grass stage. Nine genera were significantly correlated during the subtilling period: *f__Lachnospiraceae*, *s__unclassified_Pseudobutyrivibrio*, *g__Pseudobutyrivibrio*, *s__uncultured_rumen_bacterium*, *g__uncultured_rumen_bacterium*, *s__unclassified_Butyrivibrio*, *f__Muribaculaceae*, *g__Butyrivibrio*, and *o__Lachnospirales*. There were significant differences between the three groups.

**Figure 4:**
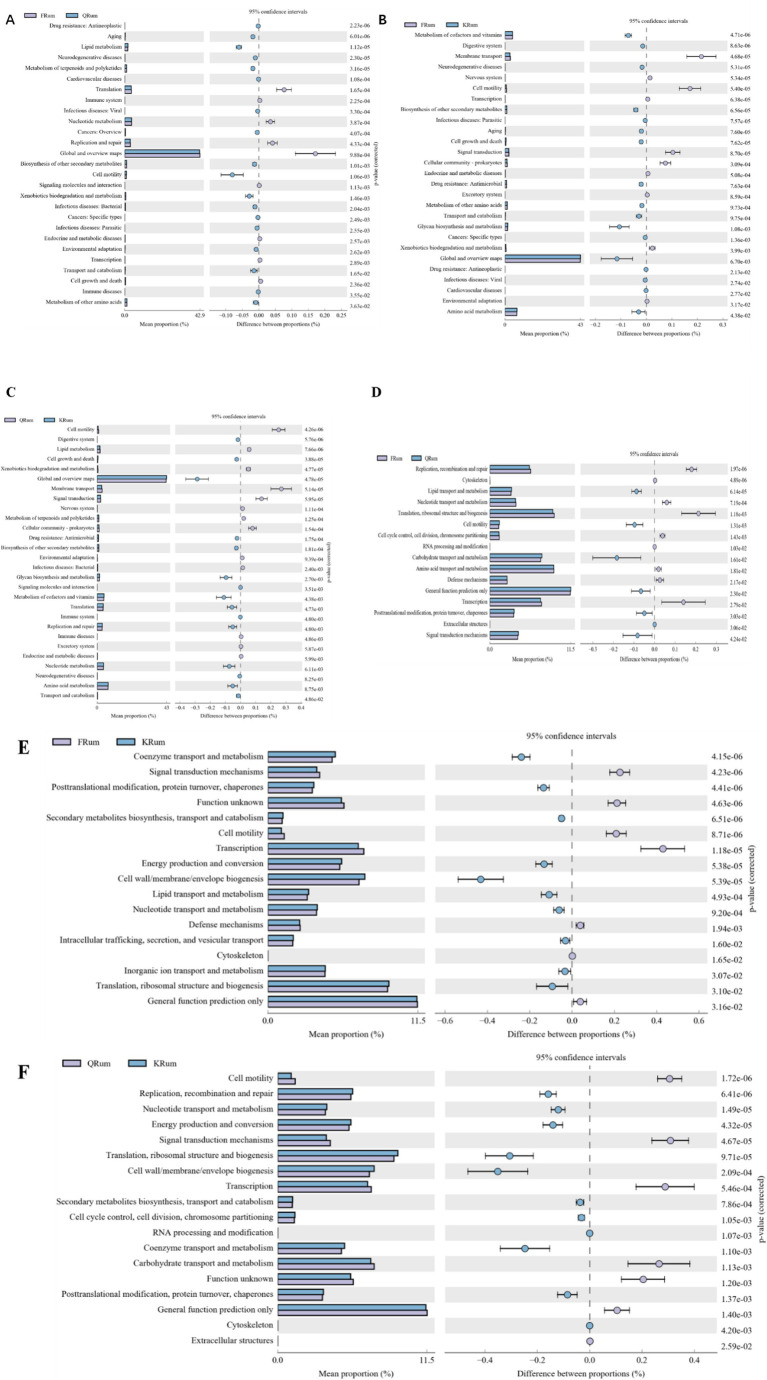
Functional analysis of rumen microorganisms in Tibetan sheep at different phenological stages. **(A)** Functional analysis of KEGG greening phase and dormant phase. **(B)** Functional analysis of KEGG vegetative phase and dormant phase. **(C)** Functional analysis of KEGG greening phase and vegetative phase. **(D)** Functional analysis of COG greening phase and dormant phase. **(E)** Functional analysis of COG vegetative phase and dormant phase. **(F)** Functional analysis of COG greening phase and vegetative phase.

PICRUSt software was used to predict gene function from 16S rRNA sequencing data, and a total of 46 KEGG gene families and 25 COG gene families were identified (Figure 4). Among them, 26 KEGG gene families and 16 COG gene families were significantly different in function. Compared with those in the hay period, membrane transport cofactors, vitamin metabolism and rumen flora signaling were significantly greater in the returning-green period. The level of amino acid metabolism was greater in the hay period than in the returning-green period. Compared with those in the returning-green period, the lipid metabolism and cellular metabolism of rumen microorganisms were greater in the hay period. However, the level of amino acid metabolism improved during the returning-green period.

### 3.5 Meat quality of the rumen microbial flora and interactions between related genes in Tibetan sheep

Correlation analysis of meat quality and related genes in Tibetan sheep at different phenophases revealed that the *FABP3* gene was significantly negatively correlated with crude fat (*p* < 0.01). The water loss rate was significantly positively correlated with the crude fat and crude protein contents (*p* < 0.01). Shear force was significantly negatively correlated with the cooked meat percentage and crude protein content (*p* < 0.05). There was no significant difference in crude fat content (*p* > 0.05). Shear force was significantly negatively correlated with crude fat and crude protein contents (*p* < 0.05). Correlation analysis of the top 10 genera and meat quality-related genes revealed that there was a correlation between the rumen microbial flora and the physical indices of meat quality and meat quality-related genes in Tibetan sheep. However, the abundance of *Butyrivibrio* was significantly positively correlated with *CAST* (*p* < 0.01). There was a significant positive correlation with crude protein and the crude fat water loss rate (*p* < 0.05) and a significant negative correlation with shear force (*p* < 0.05). The *Rikenellaceae_RC9_gut_group* was significantly negatively correlated with crude fat content (*p* < 0.01) and significantly positively correlated with the cooked meat percentage and shear force (*p* < 0.01). *Prevotella_1* was significantly negatively correlated with the crude fat and water loss rates of the *ADST* gene (*p* < 0.05) and significantly positively correlated with shear force (*p* < 0.05). The abundance of *Succiniclasticum Ruminococcus* was significantly negatively correlated with *CAST* (*p* < 0.05). *Succiniclasticum* was significantly positively correlated with *FABP3* (*p* < 0.05) and significantly negatively correlated with the water loss rate and crude protein content (*p* < 0.05). *Treponema* was significantly negatively correlated with the *ADSL* water loss rate and crude protein content (*p* < 0.05) and significantly positively correlated with shear force (*p* < 0.05).

## 4 Discussion

Meat quality is one of the main concerns of consumers when purchasing meat and is dependent mainly on the tenderness and flavor of the meat. The water loss rate and cooked meat percentage have important effects on the edible quality of livestock meat, which mainly reflects the water holding capacity of muscle, such as meat texture, flavor and juiciness (28). In this study, the water loss rate of the longest back muscle increased, and the cooked meat percentage decreased during the greening period. The water loss rate and cooked meat percentage were negatively correlated, and the lower the water loss rate was, the higher the water retention capacity of the meat was, and the higher the cooked meat percentage was, the better the meat quality was (29), indicating that the meat quality was better in the greening and dead periods than in the rejuvenation period. Meat tenderness is expressed in terms of the shear force value, which to some extent reflects muscle hardness, cohesion and elasticity (30). Pigs fed high-fiber rations have a greater advantage in terms of tenderness (31). Increased levels of ADF and NDF and lower shear values during the dry period suggest that dietary fiber plays a role in meat tenderness (32). In addition, the intramuscular fat content has a positive effect on meat juiciness and tenderness, and it was found that a 4–5% intramuscular fat content was required to satisfy Australian consumers for palatability (33). In the present study, the shear force values during the greening period were lower than those during the greening and drying periods, and the muscle fat content was approximately 3% higher than that during the other periods, suggesting that nutritional status influences the shear force of Tibetan sheep muscle, which is similar to the findings of Matthews et al. (29). Further correlation analysis between meat quality and pasture nutrients revealed that shear force was significantly negatively correlated with crude fat and crude protein contents, indicating that meat shear force values decreased as the crude fat and crude protein contents of the pasture-fed sheep increased, which may be related to the unique muscle fiber structure of Tibetan sheep and the changes in meat quality that occur during steaming. In pasture-fed lambs, the type of pasture influences the firmness of the fat cover because the proportion of legumes modulates the ratio of polyunsaturated fatty acids (PUFAs) to saturated fatty acids (SFAs) (34). Thus, grazing on legume-rich pastures results in less firm fat, an important consideration for organic farming that values legumes. pH is also an important factor in meat quality and has a direct effect on tenderness, cooking losses and shelf life (16). An accelerated glycolytic pathway facilitates ATP production and glycogen degradation in muscle after slaughter, which in turn affects the rate of pH decline and further influences meat tenderness, color and juiciness (35). In the present study, the muscle pH of the three parts ranged from 6.13 to 7.98, with a slightly acidic pH during the greening period and a neutral to slightly alkaline pH during the rejuvenation period, which may be one of the reasons for the differences in meat tenderness between the different phenological periods. Differences in pH were also found before (1 h) and after (24 h) acid drainage, with the pH at 24 h after acid drainage being significantly lower in the warm season than in the cool season, which is consistent with the results of this experiment (36). In addition to the evaluation of the abovementioned physical indicators of meat quality, chemical indicators such as moisture, ash, protein, fat, etc., are also important nutritional indicators (8). Mutton contains a high level of protein, which is very close to the protein composition of the human body and is widely preferred by consumers for its high protein and low fat characteristics compared with pork and beef. With improvements in the quality of human consumption, consumers are increasingly concerned about the fatty acids, flavor and nutritional indicators in the muscle and the changes in the nutritional composition of the muscle, especially the changes in fat and crude protein content, which directly affect the quality and nutritional characteristics of the meat (10). In this study, the moisture and crude protein contents of the hind leg muscles during the regrowth period were significantly greater than those during the grass period, whereas the crude fat content of the longest back muscle was significantly lower than that during the grass period. This result may be due to changes in muscle as a result of changes in dietary nutrients during the transition from dry to green grass, which needs to be clarified by further studies. In this study, in the grass stage, the average intake of crude protein and crude fat rose significantly from 5.71 to 19.02% and from 0.64 to 3.31%, respectively. Analysis of meat quality revealed that the muscle tenderness of Tibetan sheep improved by 10.6%, while their water retention capacity increased by 0.03%. The CP and CF contents of pasture grasses in different phenological periods tended to increase, whereas the DM content tended to decrease. The CP content was highest in the glaucous stage and lowest in the wilting stage, at 19.71 and 5.87%, respectively. In alpine pastures, grass species accumulate nutrients rapidly in the glaucous stage, and this rapid increase is related to the accumulation of nutrients in a short period of time. In addition, according to the classification of forage CP (more than 16% is considered superior, 10 to 15% is considered medium, and less than 10% is considered low) (37), the quality of forage in this experiment was medium in the green stage and low in the greening stage and the dead stage. Moreover, muscle metabolism requires catabolic fat for energy, resulting in high protein and low fat contents (38). However, these differences in meat phenotypes are also regulated at the host gene level. In this study, high expression of the *SCD* gene, a microsomal membrane-bound iron-containing enzyme required for the biosynthesis of unsaturated fatty acids, was detected in the longest muscle of the returning dorsal longus (39). *ADSL* is one of the major enzymes involved in inosine 5′-monophosphate (IMP) deposition in animals; it plays an important role in the synthesis of IMP and is closely related to the quality of meat (40). High expression of the combination of *ADSL* and *GAR-AIRS-GART* increased the content of IMP in chicken meat (41). In this study, the expression of the *ADSL* gene was significantly greater in the longest dorsal muscle during the greening period than during the greening and drying periods, which may be attributed to the nutrients and bioactive components of pasture grasses in different seasonal periods that increase the mRNA expression of the *ADSL* gene in Tibetan sheep. *CAST* and *CAPN* are correlated with muscle tenderness (42, 43). In this study, high expression of the *CAST* gene in the muscle of Tibetan sheep during the dry period led to a decrease in the expression of the *CAPN* gene, which in turn affected the tenderness of the meat, whereas Tibetan sheep during the dry period presented greater tenderness. *MSTN* is a factor that acts as a counterregulator of muscle development, and inhibition of the expression of the *MSTN* gene can lead to overproliferation of myocytes, which can result in hypertrophy of myofibers (44). *FABP3* is an important fatty acid binding protein involved in lipid metabolism and intracellular transport (45). In the correlation analysis, there was a strong correlation between water loss and crude protein content, crude fat content and shear force, and *FAST* was significantly negatively correlated with crude fat content, suggesting that meat quality is regulated by related genes.

Gastrointestinal microorganisms in ruminants are important factors affecting animal production and health, and these microorganisms also have a major influence on meat quality (46). An analysis of the rumen microorganisms of Tibetan sheep during the three seasonal periods revealed that the rumen flora of Tibetan sheep was dominated by Bacteroidetes and thick-walled bacteria at the phylum level, which is consistent with the findings of Zhang et al. (47). The Anabaena phylum is well known for its role in oligosaccharide hydrolysis and acetic and propionic acid production (48), and acetic acid plays an extremely important role in fat synthesis. However, the greater proportion of the Anabaena phylum and lower proportion of the thick-walled phylum during the dieback stage than during the regrowth and grass stages may be due to the lower rumen pH during the dieback stage, which resulted in a significantly greater proportion of the Anabaena phylum and a lower proportion of the thick-walled phylum in the rumen microbiota (49). The thick-walled phylum is involved in the degradation of cellulose, hemicellulose, starch and oligosaccharides, as well as the production of acetic, propionic and butyric acids. Although the ADF content may not differ significantly between forages, the structural properties of forages can still have a selective effect on the composition of microbial communities. The physical structure of forages, such as their fiber arrangement, cell wall thickness and cellulose crystallinity, can influence the environment for microbial attachment and growth. These structural properties can provide more suitable habitats for certain microorganisms, thereby influencing the diversity and abundance of microbial communities. For example, thick-walled bacteria may be better able to grow in environments with higher cellulose crystallinity, whereas other microbes may prefer looser fibrous structures (50). Thus, even if the ADF content is similar, microstructural differences in forages can still selectively affect microbial composition by altering microbial living conditions and nutrient access. These effects can trigger a series of chain reactions in microbial ecosystems, which in turn can affect the functioning and stability of the whole ecosystem. Nevertheless, the thick-walled bacterial phylum is likely associated with the pasture with the highest hemicellulose content and the lowest ADF content, and the ADF content did not differ in this study; thus, the changes in the thick-walled bacterial phylum may be due to differences in the hemicellulose content of the pasture. Moreover, there is a predatory relationship between rumen protozoa and bacteria, which can change the structure of the rumen microbial community. As reported by Kim et al. (51), in the phylum Bacteroidetes, the dominant genera in different phenological periods were all mainly composed of Proteobacteria and Bacteroidetes, with Proteobacteria accounting for the largest proportion, which is consistent with the results of a previous study. A similar phenomenon was observed in the present study. Bacteroidetes are closely associated with protein and carbohydrate degradation (52), and in this study, these groups were more abundant during the dieback period. In contrast, *Ruminalococcus* spp. and *Fibrobacter* spp. were enriched during the regrowth period, which may be due to their need to degrade dietary fiber and adhere to feed particles (53). KEGG functional analysis revealed that the rumen bacteria of the Tibetan sheep during the withering period were enriched in terms of cofactor and vitamin metabolism, signal transduction function and amino acid metabolism to meet the nutritional requirements during this period. Moreover, during the regrowth period, rumen microorganisms enhance lipid metabolism and cellular metabolism to help Tibetan sheep extract energy from fiber-rich pastures and promote glucose metabolism and fat accumulation (54, 55). COG functional analyses revealed that energy production and conversion, secondary metabolite biosynthesis, translocation and catabolism, and cofactor transport and metabolism were increased during the withering period. These results suggest that rumen microorganisms play an important role in energy metabolism during the withering period, while lipid metabolism was also significantly enhanced during the rejuvenation period. This result is consistent with previous studies that have shown that the fat area and myofiber density of muscle and adipose tissue increase significantly during regrowth (56). However, the underlying biological mechanisms remain to be elucidated by further research.

**Figure 5:**
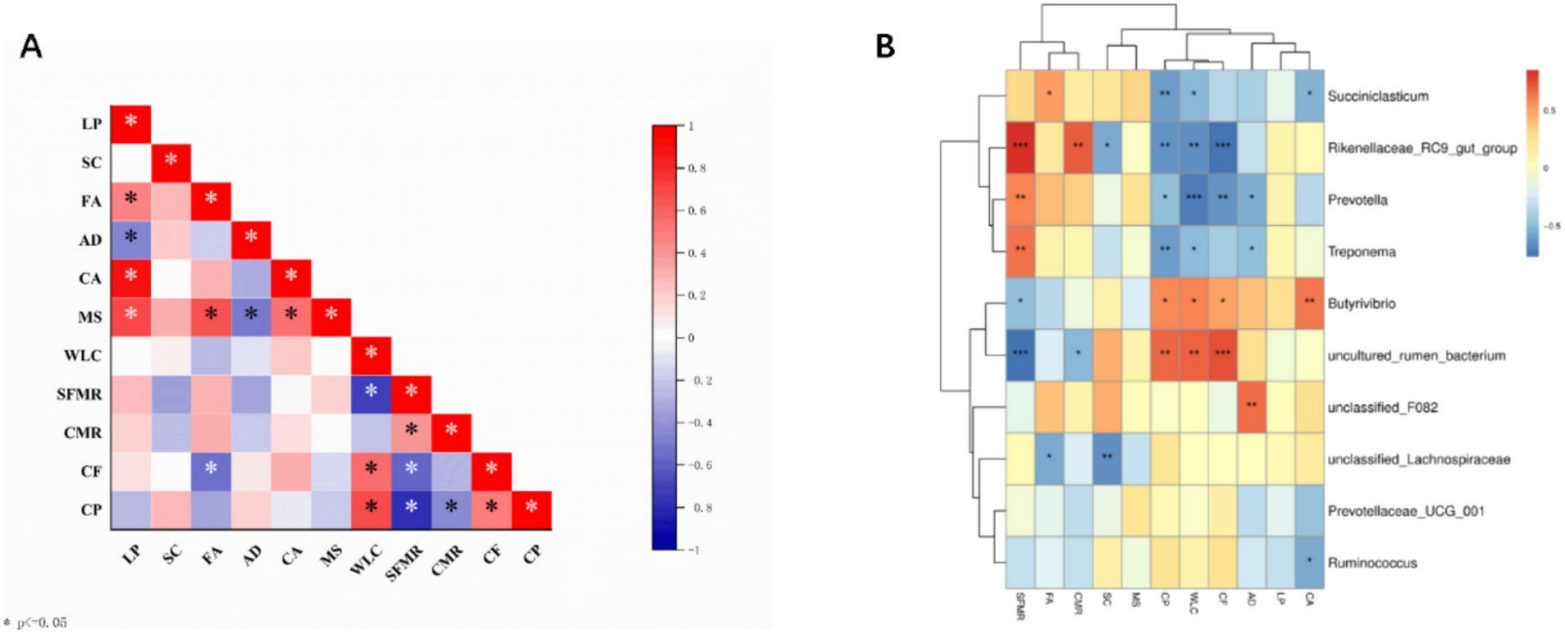
Correlation analysis of rumen microbiome, meat quality and meat quality-related genes. **(A)** Meat quality correlates with gene expression. **(B)** Microbial correlation with meat quality and related gene expression. LP (LPL); SC (SCD); FA (FABP3); AD (ADSL); CA (CAST); MS (MSTN); water loss rate (WLC); shear force (SFMR); cooked meat percentage (CMR); crude fat (CF); crude protein (CP); ^*^p < 0.05, ^**^p < 0.01, and ^***^p < 0.001.

**Figure 6:**
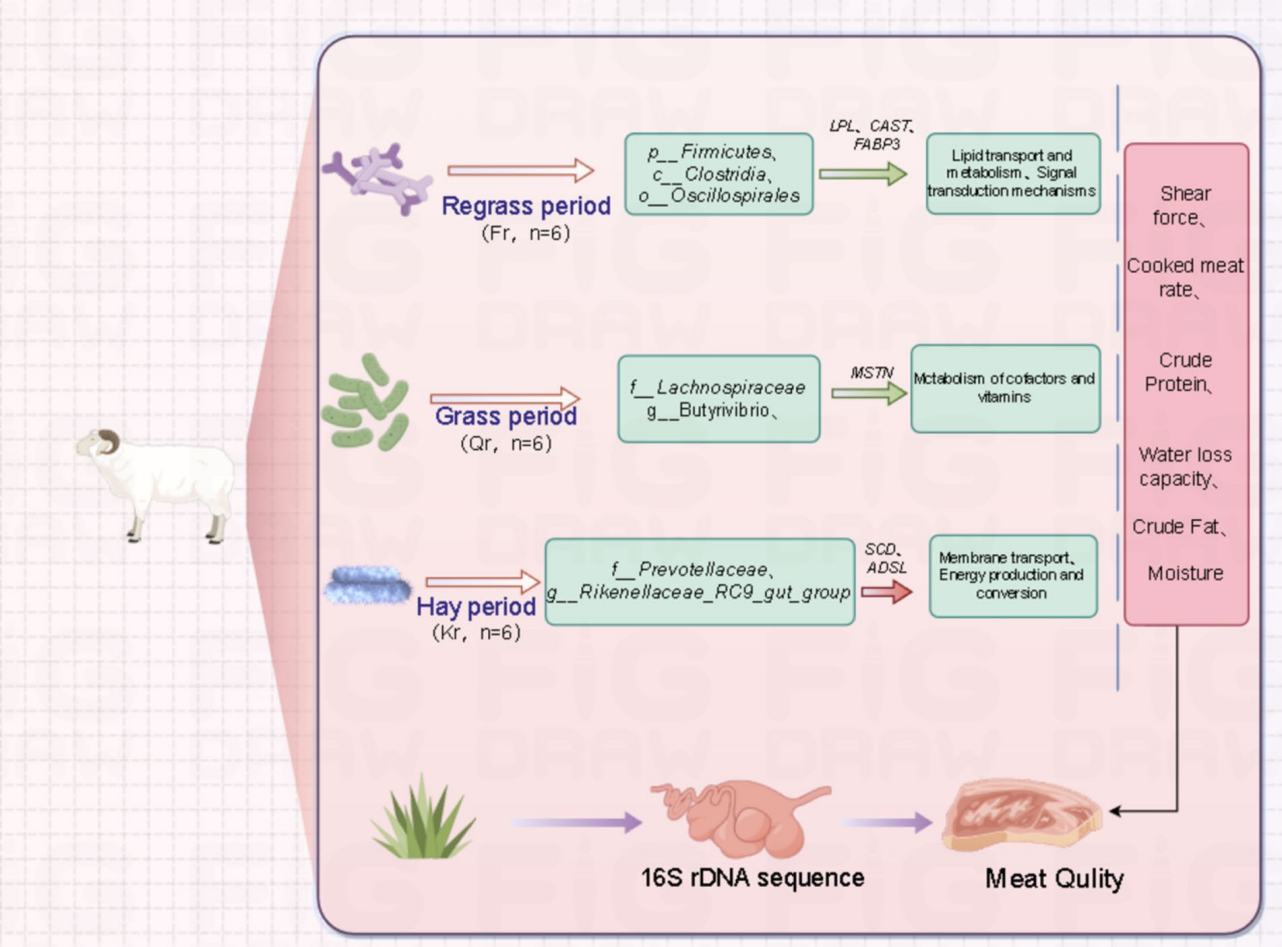
Regulation of rumen flora and meat quality related genes of Tibetan sheep at different phenological stages on meat quality. Green solid arrowhead: positive correlation. Red solid arrowhead: negative correlation.

In addition, an association analysis of the rumen microbiota with meat quality indicators and their associated genes (Figure 5) was performed in this study, and the top 10 genus-level microbiota were selected for association with meat quality phenotypic traits and their associated genes. *Rikenellaceae_RC9_gut_group* and *Prevotella* spp. were significantly associated with crude fat. It has been shown that the *Rikenellaceae_RC9_gut_group* can affect lipid and glucose metabolism in grazing sheep by promoting the deposition of glycodeoxycholic acid, α-linolenic acid and glycolenylcholic acid (57). The *Rikenellaceae_RC9_gut_group* was significantly negatively correlated with the *SCD* gene, which in the animal body is mainly responsible for the conversion of saturated fatty acids to monounsaturated fatty acids (58), a process important for improving meat quality, and the *Rikenellaceae_RC9_gut_group* and *Prevotella* were the dominant genera in the wilting period. In addition, *Prevotella* abundance was significantly negatively correlated with crude protein content and shear strength. It has been reported that *Prevotella* possesses enzymes and gene clusters necessary for the fermentation and utilization of complex polysaccharides and is able to efficiently degrade proteins and carbohydrates, which in turn leads to the synthesis of microbial proteins (59). The important role of *Prevotella* in the rumen ecosystem and its effect on meat formation were confirmed. *Succiniclasticum* spp. was significantly negatively correlated with *CAST*, significantly positively correlated with *FABP3* and significantly negatively correlated with water loss and crude protein content. As the main acid-producing bacteria of succinic acid in the rumen, *Succiniclasticum* plays a key role in the conversion of succinic acid to propionic acid (60). The conversion of propionic acid to glucose (GLU) in the liver via gluconeogenesis in the rumen wall is important during the fattening period of Tibetan sheep (61). In the present study, *Ruminococcus* spp. and *Butyrivibrio* spp. were highly significantly positively correlated with *CAST* genes, and *Butyrivibrio_2* was significantly associated with fatty acids in meat (62), which may play a role in butyric acid production (63). In the present study, dense spirochetes (*Treponema*) were significantly negatively correlated with the *ADSL* gene, water loss and crude protein content and significantly positively correlated with shear strength. A possible explanation is that these dense spirochete species, which are unable to utilize cellulose, may have established a strong synergistic relationship with cellulose-degrading bacteria to obtain nutrients by utilizing the soluble sugars released during fiber degradation (64). Dense spirochetes with low cellulose degradation capacity have been identified in sheep rumen, suggesting that they may assist other bacteria in the utilization of cellulose substrates (65). In previous functional analyses of KEGG and COG, it was observed that rumen microflora across different phenological stages regulate energy utilization mechanisms by influencing pathways related to lipid, energy, and amino acid metabolism within the host’s metabolic framework. This, in turn, impacts meat quality. Beneficial rumen bacteria are intricately linked with metabolites, such as amino acids and fatty acids, which are crucial to meat nutrients, playing a significant role in lipid metabolism (28). Our study found a high relative abundance of bacteria associated with lipid metabolism (e.g., *o__Oscillospirales*) and beneficial functions (*Lactobacillus* and *Clostridium*) (65), further substantiating our hypothesis. By integrating 16S rDNA sequences with fleshy and related genes, we uncovered novel relationships between rumen bacteria and genes associated with meat characteristics (Figure 6). These findings offer the potential to enhance the feeding system of Tibetan sheep on the Tibetan Plateau, thereby improving the quality of sheep meat.

Modifications in the nutrient profile of pasture significantly influence the structure and functionality of the rumen microbial community across various phenological stages, with profound implications for meat quality. During the early growth phase, pasture grasses exhibit high protein content. Consumption of these high-protein feeds not only enhances muscle protein synthesis and improves meat tenderness but may also alter the rumen microbial community’s composition and metabolic activity through increased ammonia production (15). These changes in the rumen microbiota can affect the host’s metabolic state and the expression of genes related to fatty acid synthesis and muscle fiber types. As pasture progresses to maturity, its fiber content rises, potentially reducing the animal’s digestive efficiency and consequently impacting meat quality (66). The rumen microbial community exhibits adaptability, allowing it to adjust its composition to better handle the challenges posed by high-fibre diets. This adaptability can influence meat tenderness by affecting gene expression and influencing muscle growth and fibre type. Additionally, the phenological stage of pasture consumption affects the growth rate and metabolic state of the animal, which is closely linked to changes in muscle fibre type (67, 68). For example, high-protein diets can promote the development of fast-growing muscle fibre types, thus improving meat tenderness. These changes not only illustrate the direct impact of nutrients on meat quality but also underscore the roles of rumen microbial communities and gene expression in meat quality regulation. Ultimately, the consumption of pasture at different phenological stages leads to variations in meat quality due to changes in nutrient composition, rumen microbial communities, and associated gene expression. These findings suggest new avenues for optimizing meat quality and highlight the need for comprehensive studies on the interactions between forage nutrient composition, microbial communities, and gene expression.

## 5 Conclusion

This study aimed to elucidate the effects of different seasonal periods on meat quality, the microbiota and related gene expression in Tibetan sheep. The analysis of Tibetan sheep during three seasonal periods revealed that changes in the seasonal period not only directly affected meat quality in terms of water holding capacity and cookability but also closely correlated with the levels of nutrients, such as CF and CP, in the meat. Notably, grazing during the season modulates the meat quality of Tibetan sheep by influencing the composition of the rumen microbial community. This mechanism is highly dependent on microbial effects on the expression of relevant genes. In particular, during the dry period, the expression level of the *ADSL* gene was reduced in the hind leg muscles, which was directly related to changes in meat fat content. Conversely, high expression of the *CAST* gene led to changes in meat tenderness, which manifested as a reduction in muscle shear force values. These alterations in gene expression were closely associated with variations in rumen microbial communities across different phenological stages. The microbial community regulates the rumen environment at different times, which in turn affects the expression of genes related to fatty acid metabolism and meat tenderness and thus influences meat quality traits. In conclusion, this study elucidated the important regulatory effects of pasture on the rumen microbial composition of Tibetan sheep during different phenological periods. This study not only provides insights at the gene level for understanding meat quality changes but also provides a scientific basis for enhancing lamb meat quality and improving feeding management strategies. Further studies could investigate the potential for optimizing meat quality through adjustments to pasture cropping strategies or microbial supplementation interventions.

## Data Availability

The data sets presented in this study can be found in the NCBI Sequence Read Archive (SRA) under accession numbers PRJNA1135556.

## References

[1] DeWitt PD Schuler MS Visscher DR Thiel RP. Nutritional state reveals complex consequences of risk in a wild predator–prey community. Proc Biol Sci. (2017) 284:20170757. doi: 10.1098/rspb.2017.0757 28701562 PMC5524499

[2] Lv W Liu X Sha Y Shi H Wei H Luo Y. Rumen fermentation—microbiota—host gene expression interactions to reveal the adaptability of Tibetan sheep in different periods. Animals. (2021) 11:3529. doi: 10.3390/ani11123529, PMID: 34944301 PMC8697948

[3] Yang C Gao P Hou F Yan T Chang S Chen X. Relationship between chemical composition of native forage and nutrient digestibility by Tibetan sheep on the Qinghai–Tibetan Plateau. J Anim Sci. (2018) 96:1140–9. doi: 10.1093/jas/sky002, PMID: 29617805 PMC6140931

[4] Zha XJ Tian Y Ouzhu FG. Response of forage nutrient storages to grazing in alpine grasslands. Front Plant Sci. (2022) 13:991287. doi: 10.3389/fpls.2022.991287, PMID: 36388576 PMC9664390

[5] Liu H Hu L Han X Zhao N Xu T Ma L. Tibetan sheep adapt to plant phenology in alpine meadows by changing rumen microbial community structure and function. Front Microbiol. (2020) 11:587558. doi: 10.3389/fmicb.2020.587558, PMID: 33193243 PMC7649133

[6] Jing XP Wang WJ Degen AA Guo YM Kang JP Liu PP. Small intestinal morphology and sugar transporters expression when consuming diets of different energy levels: comparison between Tibetan and small—tailed Han sheep. Animal. (2022) 16:100463. doi: 10.1016/j.animal.2022.100463, PMID: 35193064

[7] Liu X Sha Y Lv W Cao G Guo X Pu X. Multi-omics reveals that the rumen transcriptome, microbiome, and its metabolome co-regulate cold season adaptability of Tibetan sheep. Front Microbiol. (2022) 13:859601. doi: 10.3389/fmicb.2022.859601, PMID: 35495720 PMC9043902

[8] Zhang X Han L Hou S Raza SHA Gui L Sun S. Metabolomics approach reveals high energy diet improves the quality and enhances the flavor of black Tibetan sheep meat by altering the composition of rumen microbiota. Front Nutr. (2022) 9:915558. doi: 10.3389/fnut.2022.915558, PMID: 36034898 PMC9405419

[9] Prache S Schreurs N Guillier L. Review: factors affecting sheep carcass and meat quality attributes. Animal. (2022) 16:100330. doi: 10.1016/j.animal.2021.100330, PMID: 34400114

[10] Moon SS Yang HS Park GB Joo ST. The relationship of physiological maturity and marbling judged according to Korean grading system to meat quality traits of Hanwoo beef females. Meat Sci. (2006) 74:516–21. doi: 10.1016/j.meatsci.2006.04.027, PMID: 22063056

[11] Yang Y Wang Y Shan H Zheng Y Xuan Z Hu J. Novel insights into the differences in nutrition value, gene regulation and network organization between muscles from pasture-fed and barn-fed goats. Foods. (2022) 11:381. doi: 10.3390/foods11030381 35159531 PMC8834483

[12] Steelman SM Chowdhary BP Dowd S Suchodolski J Janecka JE. Pyrosequencing of 16S rRNA genes in fecal samples reveals high diversity of hindgut microflora in horses and potential links to chronic laminitis. BMC Vet Res. (2012) 8:231. doi: 10.1186/1746-6148-8-231 23186268 PMC3538718

[13] Zhou J Xue B Hu A Yue S Wu M Hong Q. Effect of dietary peNDF levels on digestibility and rumen fermentation, and microbial community in growing goats. Front Microbiol. (2022) 13:950587. doi: 10.3389/fmicb.2022.950587, PMID: 36090059 PMC9453810

[14] Matthews C Crispie F Lewis E Reid M O’Toole PW Cotter PD. The rumen microbiome: a crucial consideration when optimising milk and meat production and nitrogen utilisation efficiency. Gut Microbes. (2019) 10:115–32. doi: 10.1080/19490976.2018.1505176 30207838 PMC6546327

[15] Li L Sun X Luo J Chen T Xi Q Zhang Y. Effects of herbal tea residue on growth performance, meat quality, muscle metabolome, and rumen microbiota characteristics in finishing steers. Front Microbiol. (2021) 12:821293. doi: 10.3389/fmicb.2021.821293 35116016 PMC8804378

[16] Zhu X Liu B Xiao J Guo M Zhao S Hu M. Effects of different roughage diets on fattening performance, meat quality, fatty acid composition, and rumen microbe in steers. Front Nutr. (2022) 9:885069. doi: 10.3389/fnut.2022.885069, PMID: 35799586 PMC9253607

[17] Qin X Zhang D Qiu X Zhao K Zhang S Liu C. 2-hydroxy-4-(methylthio) butanoic acid isopropyl ester supplementation altered ruminal and cecal bacterial composition and improved growth performance of finishing beef cattle. Front Nutr. (2022) 9:833881. doi: 10.3389/fnut.2022.833881, PMID: 35600827 PMC9116427

[18] Association of Official Analytical Chemists (AOAC). Official methods of analysis. 16th ed. Washington, DC: AOAC International (1997).

[19] Van Soest PJ Robertson JB Lewis BA. Methods for dietary fiber, neutral detergent fiber, and nonstarch polysaccharides in relation to animal nutrition. J Dairy Sci. (1991) 74:3583–97. doi: 10.3168/jds.S0022-0302(91)78551-2, PMID: 1660498

[20] Devapriya A Sejian V Ruban W Devaraj C Spandan PV Silpa MV. Analysis of carcass traits and quantitative expression patterns of different meat quality governing genes during heat stress exposure in indigenous goats. Food Chem. (2021) 3:100052. doi: 10.1016/j.fochms.2021.100052, PMID: 35415654 PMC8991526

[21] Atsbha K Gebremariam T Aregawi T. Slaughter performance and meat quality of Begait breed lambs fattened under different diets. Heliyon. (2021) 7:e6935. doi: 10.1016/j.heliyon.2021.e06935, PMID: 34013082 PMC8113829

[22] Honikel KO. Reference methods for the assessment of physical characteristics of meat. Meat Sci. (1998) 49:447–57. doi: 10.1016/S0309-1740(98)00034-5, PMID: 22060626

[23] Association of Official Analytical Chemists (AOAC). Errata and emendations, Official Methods of Analysis, AOAC. J AOAC Int. (1971) 54:497. doi: 10.1093/jaoac/54.2.497, PMID: 649556

[24] Livak KJ Schmittgen TD. Analysis of relative gene expression data using real-time quantitative PCR and the $2^{-\Delta \Delta C_{T}}$ method. Methods. (2001) 25:402–8. doi: 10.1006/meth.2001.1262 11846609

[25] Callahan BJ McMurdie PJ Rosen MJ Han AW Johnson AJ Holmes SP. DADA2: high-resolution sample inference from Illumina amplicon data. Nat Methods. (2016) 13:581–3. doi: 10.1038/nmeth.3869 27214047 PMC4927377

[26] Bolyen E Rideout JR Dillon MR Bokulich NA Abnet CC Al-Ghalith GA. Reproducible, interactive, scalable and extensible microbiome data science using QIIME 2. Nat Biotechnol. (2019) 37:852–7. doi: 10.1038/s41587-019-0209-9 31341288 PMC7015180

[27] Quast C Pruesse E Yilmaz P Gerken J Schweer T Yarza P. The SILVA ribosomal RNA gene database project: improved data processing and web-based tools. Nucleic Acids Res. (2013) 41:D590–6. doi: 10.1093/nar/gks1219, PMID: 23193283 PMC3531112

[28] Ma Y Han L Zhang S Zhang X Hou S Gui L. Insight into the differences of meat quality between Qinghai white Tibetan sheep and black Tibetan sheep from the perspective of metabolomics and rumen microbiota. Food Chem X. (2023) 19:100843. doi: 10.1016/j.fochx.2023.100843, PMID: 37780244 PMC10534161

[29] Matthews JO Higbie AD Southern LL Coombs DF Bidner TD Odgaard RL. Effect of chromium propionate and metabolizable energy on growth, carcass traits, and pork quality of growing-finishing pigs. J Anim Sci. (2003) 81:191–6. doi: 10.2527/2003.811191x 12597390

[30] Xu X Wei Y Zhang Y Jing X Cong X Gao Q. A new selenium source from Se-enriched *Cardamine violifolia* improves growth performance, anti-oxidative capacity and meat quality in broilers. Front Nutr. (2022) 9:996932. doi: 10.3389/fnut.2022.996932, PMID: 36105580 PMC9465325

[31] Comi G Muzzin A Corazzin M Iacumin L. Lactic acid bacteria: variability due to different pork breeds, breeding systems and fermented sausage production technology. Foods. (2020) 9:338. doi: 10.3390/foods9030338, PMID: 32183247 PMC7142627

[32] Huang CH Lin CS Lee YC Ciou JW Kuo CH Huang CY. Quality improvement in mackerel fillets caused by brine salting combined with high-pressure processing. Biology. (2022) 11:1307. doi: 10.3390/biology11091307, PMID: 36138786 PMC9495997

[33] Tian F Decker EA Goddard JM. Controlling lipid oxidation of food by active packaging technologies. Food Funct. (2013) 4:669–80. doi: 10.1039/c3fo30360h 23576007

[34] Lourenço M Van Ranst G De Smet S. Effect of grazing pastures with different botanical composition by lambs on rumen fatty acid metabolism and fatty acid pattern of longissimus muscle and subcutaneous fat. Animal. (2007) 1:537–45. doi: 10.1017/S1751731107703531, PMID: 22444411

[35] Chen C Guo Z Shi X Guo Y Ma G Ma J. H_2_O_2_-induced oxidative stress improves meat tenderness by accelerating glycolysis via hypoxia-inducible factor-1α signaling pathway in postmortem bovine muscle. Food Chem X. (2022) 16:100466. doi: 10.1016/j.fochx.2022.100466, PMID: 36225213 PMC9550526

[36] Sha Y He Y Liu X Shao P Wang F Xie Z. Interactions of rumen microbiota and metabolites with meat quality-related genes to regulate meat quality and flavor of Tibetan sheep under nutrient stress in the cold season. J Appl Microbiol. (2023) 134:lxad182. doi: 10.1093/jambio/lxad182, PMID: 37567778

[37] Fan X Xuan C Zhang M Ma Y Meng Y. Estimation of spatial-temporal distribution of grazing intensity based on sheep trajectory data. Sensors. (2022) 22:1469. doi: 10.3390/s22041469, PMID: 35214370 PMC8875336

[38] Bu Z Ge G Jia Y Du S. Effect of hay with or without concentrate or pellets on growth performance and meat quality of Ujimqin lambs on the Inner Mongolian Plateau. Anim Sci J. (2021) 92:e13553. doi: 10.1111/asj.13553, PMID: 33938599

[39] Enoch HG Catala A Strittmatter P. Mechanism of rat liver microsomal stearyl-CoA desaturase. Studies of the substrate specificity, enzyme-substrate interactions, and the function of lipid. J Biol Chem. (1976) 251:5095–103. 8453

[40] Mao HG Cao HY Liu HH Dong XY Xu NY Yin ZZ. Association of ADSL gene polymorphisms with meat quality and carcass traits in domestic pigeons (*Columba livia*). Br Poultry Sci. (2018) 59:604–7. doi: 10.1080/00071668.2018.1493188, PMID: 29963908

[41] Shu JT Bao WB Zhang XY Ji CJ Han W Chen KW. Combined effect of mutations in ADSL and GARS-AIRS-GART genes on IMP content in chickens. Br Poultry Sci. (2009) 50:680–6. doi: 10.1080/00071660903391709 19946821

[42] Sun X Wu X Fan Y Mao Y Ji D Huang B. Effects of polymorphisms in *CAPN1* and *CAST* genes on meat tenderness of Chinese Simmental cattle. Arch Anim Breed. (2018) 61:433–9. doi: 10.5194/aab-61-433-2018, PMID: 32175450 PMC7065412

[43] Pinto LF Ferraz JB Pedrosa VB Eler JP Meirelles FV Bonin MN. Single nucleotide polymorphisms in CAPN and leptin genes associated with meat color and tenderness in Nellore cattle. Genet Mol Res. (2011) 10:2057–64. doi: 10.4238/vol10-3gmr1263, PMID: 21968622

[44] He N Lang X Wang C Lv C Li M Sun R. Expression of MSTN/Smad signaling pathway genes and its association with meat quality in Tibetan sheep (*Ovis aries*). Food Sci Nutr. (2023) 11:1836–45. doi: 10.1002/fsn3.3216, PMID: 37051366 PMC10084970

[45] Mao HG Xu XL Cao HY Dong XY Zou XT Xu NY. H-FABP gene expression and genetic association with meat quality traits in domestic pigeons (*Columba livia*). Br Poultry Sci. (2021) 62:172–9. doi: 10.1080/00071668.2020.1839016, PMID: 33174489

[46] Jing C Wang J Xie Y Zhang J Guo Y Tian T. Investigation of the growth performance, blood status, gut microbiome and metabolites of rabbit fed with low-nicotine tobacco. Front Microbiol. (2022) 13:1026680. doi: 10.3389/fmicb.2022.1026680, PMID: 36312940 PMC9615924

[47] Zhang YK Zhang XX Li FD Li C Li GZ Zhang DY. Characterization of the rumen microbiota and its relationship with residual feed intake in sheep. Animal. (2021) 15:100161. doi: 10.1016/j.animal.2020.100161 33785185

[48] Chen XJ Mao HL Ma XM Liu JX. Effects of dietary corn oil and vitamin E supplementation on fatty acid profiles and expression of acetyl CoA carboxylase and stearoyl-CoA desaturase gene in Hu sheep. Anim Sci J. (2010) 81:165–71. doi: 10.1111/j.1740-0929.2009.00731.x 20438496

[49] Hook SE Steele MA Northwood KS Dijkstra J France J Wright AD. Impact of subacute ruminal acidosis (SARA) adaptation and recovery on the density and diversity of bacteria in the rumen of dairy cows. FEMS Microbiol Ecol. (2011) 78:275–84. doi: 10.1111/j.1574-6941.2011.01154.x 21692816

[50] Chen Y Wang X Wang X Cheng T Fu K Qin Z. Biofilm structural and functional features on microplastic surfaces in greenhouse agricultural soil. Sustainability. (2022) 14:7024. doi: 10.3390/su14127024, PMID: 39659294

[51] Kim M Morrison M Yu Z. Status of the phylogenetic diversity census of ruminal microbiomes. FEMS Microbiol Ecol. (2011) 76:49–63. doi: 10.1111/j.1574-6941.2010.01029.x 21223325

[52] Xue MY Sun HZ Wu XH Liu JX Guan LL. Multi-omics reveals that the rumen microbiome and its metabolome together with the host metabolome contribute to individualized dairy cow performance. Microbiome. (2020) 8:64. doi: 10.1186/s40168-020-00819-8 32398126 PMC7218573

[53] McAllister TA Bae HD Jones GA Cheng KJ. Microbial attachment and feed digestion in the rumen. J Anim Sci. (1994) 72:3004–18. doi: 10.2527/1994.72113004x 7730196

[54] Yu J Liang F Huang H Pirttiniemi P Yu D. Effects of loading on chondrocyte hypoxia, HIF-1alpha and VEGF in the mandibular condylar cartilage of young rats. Orthod Craniofac Res. (2018) 21:41–7. doi: 10.1111/ocr.12212, PMID: 29271061

[55] Kovatcheva-Datchary P Nilsson A Akrami R Lee YS De Vadder F Arora T. Dietary fiber-induced improvement in glucose metabolism is associated with increased abundance of *Prevotella*. Cell Metab. (2015) 22:971–82. doi: 10.1016/j.cmet.2015.10.001 26552345

[56] Jang D Yoon J Taye M Lee W Kwon T Shim S. Multivariate genome-wide association studies on tenderness of Berkshire and Duroc pig breeds. Genes Genomics. (2018) 40:701–5. doi: 10.1007/s13258-018-0672-6 29934809

[57] Wang B Wang Y Zuo S Peng S Wang Z Zhang Y. Untargeted and targeted metabolomics profiling of muscle reveals enhanced meat quality in artificial pasture grazing tan lambs via rescheduling the rumen bacterial community. J Agric Food Chem. (2021) 69:846–58. doi: 10.1021/acs.jafc.0c06427, PMID: 33405917

[58] Poklukar K Candek-Potokar M Vrecl M Batorek-Lukac N Fazarinc G Kress K. Adipose tissue gene expression of entire male, immunocastrated and surgically castrated pigs. Int J Mol Sci. (2021) 22:1768. doi: 10.3390/ijms22041768, PMID: 33578947 PMC7916650

[59] Chen D Jin D Huang S Wu J Xu M Liu T. *Clostridium butyricum*, a butyrate-producing probiotic, inhibits intestinal tumor development through modulating Wnt signaling and gut microbiota. Cancer Lett. (2020) 469:456–67. doi: 10.1016/j.canlet.2019.11.019 31734354

[60] Ahmad AA Yang C Zhang J Kalwar Q Liang Z Li C. Effects of dietary energy levels on rumen fermentation, microbial diversity, and feed efficiency of yaks (*Bos grunniens*). Front Microbiol. (2020) 11:625. doi: 10.3389/fmicb.2020.00625, PMID: 32670204 PMC7326093

[61] Conte G Dimauro C Daghio M Serra A Mannelli F McAmmond BM. Exploring the relationship between bacterial genera and lipid metabolism in bovine rumen. Animal. (2022) 16:100520. doi: 10.1016/j.animal.2022.100520 35468508

[62] Baldwin RT Wu S Li W Li C Bequette BJ Li RW. Quantification of transcriptome responses of the rumen epithelium to butyrate infusion using RNA-seq technology. Gene Regul Syst Biol. (2012) 6:67–80. doi: 10.4137/GRSB.S9687 PMC3362330 22654504

[63] Kudo H Cheng KJ Costerton JW. Interactions between *Treponema bryantii* and cellulolytic bacteria in the *in vitro* degradation of straw cellulose. Can J Microbiol. (1987) 33:244–8. doi: 10.1139/m87-041 3567744

[64] Nyonyo T Shinkai T Mitsumori M. Improved culturability of cellulolytic rumen bacteria and phylogenetic diversity of culturable cellulolytic and xylanolytic bacteria newly isolated from the bovine rumen. FEMS Microbiol Ecol. (2014) 88:528–37. doi: 10.1111/1574-6941.12318 24612331

[65] Qi K Men X Wu J Xu Z. Rearing pattern alters porcine myofiber type, fat deposition, associated microbial communities and functional capacity. BMC Microbiol. (2019) 19:181. doi: 10.1186/s12866-019-1556-x 31387544 PMC6683424

[66] Sakowski T Grodkowski G Golebiewski M Slosarz J Kostusiak P Solarczyk P. Genetic and environmental determinants of beef quality-a review. Front Vet Sci. (2022) 9:819605. doi: 10.3389/fvets.2022.819605, PMID: 35280136 PMC8907586

[67] Perry EB Valach AA Fenton JM Moore GE. An assessment of starch content and gelatinization in traditional and non-traditional dog food formulations. Animals. (2022) 12:3357. doi: 10.3390/ani12233357, PMID: 36496878 PMC9739134

[68] Yuan Z Ge L Zhang W Lv X Wang S Cao X. Preliminary results about lamb meat tenderness based on the study of novel isoforms and alternative splicing regulation pathways using Iso-seq, RNA-seq and CTCF ChIP-seq data. Foods. (2022) 11:1068. doi: 10.3390/foods11081068, PMID: 35454655 PMC9025809

